# Speech Intelligibility in Reverberation is Reduced During Self-Rotation

**DOI:** 10.1177/23312165231188619

**Published:** 2023-07-20

**Authors:** Ľuboš Hládek, Bernhard U. Seeber

**Affiliations:** 1 Audio Information Processing, 9184 Technical University of Munich, Munich, Germany

**Keywords:** speech understanding, head rotation, spatial unmasking, speech intelligibility model

## Abstract

Speech intelligibility in cocktail party situations has been traditionally studied for stationary sound sources and stationary participants. Here, speech intelligibility and behavior were investigated during active self-rotation of standing participants in a spatialized speech test. We investigated if people would rotate to improve speech intelligibility, and we asked if knowing the target location would be further beneficial. Target sentences randomly appeared at one of four possible locations: 0°, ± 90°, 180° relative to the participant's initial orientation on each trial, while speech-shaped noise was presented from the front (0°). Participants responded naturally with self-rotating motion. Target sentences were presented either without (Audio-only) or with a picture of an avatar (Audio–Visual). In a baseline (Static) condition, people were standing still without visual location cues. Participants’ self-orientation undershot the target location and orientations were close to acoustically optimal. Participants oriented more often in an acoustically optimal way, and speech intelligibility was higher in the Audio–Visual than in the Audio-only condition for the lateral targets. The intelligibility of the individual words in Audio–Visual and Audio-only increased during self-rotation towards the rear target, but it was reduced for the lateral targets when compared to Static, which could be mostly, but not fully, attributed to changes in spatial unmasking. Speech intelligibility prediction based on a model of static spatial unmasking considering self-rotations overestimated the participant performance by 1.4 dB. The results suggest that speech intelligibility is reduced during self-rotation, and that visual cues of location help to achieve more optimal self-rotations and better speech intelligibility.

## Introduction

At cocktail parties, people are often moving. In conversations, people come closer to each other and increase their voices when there is a high level of background noise (Beechey et al., 2018; Cheyne et al., 2009; Hadley et al., 2019; Latif et al., 2014). In some situations, people make sudden and more prominent movements during listening. For instance, if somebody new enters the scene from a side or from behind and is addressing the group, then people naturally turn towards the person to see them and listen to what they are saying. This is a common situation but previous research on speech perception often neglected the effect of self-movement on speech intelligibility. This is particularly relevant for research on hearing aids because it has been suggested that movement is a possible limiting factor for hearing aid benefit in real-world situations (Bentler, 2005; Ching et al., 2009; Cord et al., 2004). Numerous studies aimed to create ecologically valid and individualized assessments of hearing abilities in realistic scenarios (Beechey et al., 2018; Best et al., 2007; Brungart et al., 2020; Gonzalez-Franco et al., 2017; Hendrikse et al., 2018, 2019; Kerber & Seeber, 2011, 2013; Kishline et al., 2020; Stecker, 2019; Viveros Muñoz et al., 2019), however, they did not cover situations when people make pronounced turns and listen to speech at the same time.

Research traditionally finds that people with normal hearing are exceptionally good at understanding speech at cocktail parties. One of the prominent factors is spatial separation of the target from the interferer—spatial unmasking (Bronkhorst, 2000, 2015; Culling et al., 2004). Here, we will refer to spatial unmasking as an acoustic and auditory phenomenon. When a sound travels towards a listener, the head creates an acoustic shadow, which improves signal-to-noise ratio (SNR) at one of the ears, hence one component of spatial unmasking is ‘better ear listening’, while the other component, ‘binaural unmasking’, is considered a contribution of the binaural system to improve detection of the target in the interferer. Tests of spatial unmasking are traditionally conducted for static sounds and static participants; however, less is known about how these benefits translate to moving people or sources (Bronkhorst, 2000, 2015).

Previous results suggested that people may adapt their head movements for better intelligibility, but it depends on the pose (Hendrikse et al., 2019), the SNR (Brimijoin et al., 2012), the instructions (Grange et al., 2018), the visual cues (Hendrikse et al., 2018) and possibly other attention and experience-related factors (Best et al., 2008; Kidd et al., 2005) (see review by Grimm et al. (2020)). Brimijoin et al. (2012) used a spatialized speech-in-noise test with unrestricted head movements. They used short sentences as targets and a single noise interferer with varying position. They evaluated the head rotations using motion tracking data and a modified version of a speech intelligibility model (Jelfs et al., 2011). The experimenters expected that people would adapt head rotations for better understanding. Instead, they observed that people always maintained an off-target horizontal head orientation. Therefore, participants effectively ignored the positional changes of the interferer, suggesting that movement-induced changes in intelligibility did not play a critical role in behavior. Grange and Culling (2016) investigated head movement behavior of sitting participants in a listening task with unrestricted head movements, in which the noise level decreased progressively (7.5 dB/min) until participants lost track of the running target speech (speech of a politician). The experimenters observed high variability in head orienting movements since the propensity to head movements varied between participants (e.g., some participants did not move), and thus only a weak contribution of head motion to speech intelligibility. However, the instructions turned out to be a critical factor for people using head movements to improve speech intelligibility, which they elaborated in a later study. Grange et al. (2018) showed that the participants improved their speech reception thresholds when they were properly instructed to use head movements. High variance in propensity to head movements was also observed in an experiment in which the participants were instructed to follow speech signals originating at two locations in pseudo-random intervals (Hládek et al., 2019), which suggested that people used different strategies of head movements in cocktail party situations.

Hendrikse et al. (2018) conducted two experiments with audio-visual stimuli. In the first one, sitting participants followed a spatialized pre-recorded conversation with unrestricted head movements. In the second one, which was a spatialized speech-in-noise test, the participants were instructed to attend to the speaker (indicated by a call sign) and then localize it. The experimenters manipulated the presence and quality of visual cues of the talking avatars by giving them lip movements or letting them look at the current talker, participant, or at a random direction. In the first experiment, most participants did not move their head when no avatars were present. In other conditions, with avatars present, the participants usually looked at the currently speaking avatar, but the participants’ horizontal head orientation did not point directly at the target but maintained an off-target horizontal direction. In the second experiment, when the other avatars looked at the currently speaking avatar, the performance of the observing participant, measured in terms of combined localization and speech perception, improved, but speech perception alone was not analyzed. Overall, both experiments showed that the visual features influence the pattern of head rotations and gaze movements. However, the study did not identify a consistent strategy to optimize speech intelligibility by means of head movements.

While the abovementioned studies considered head rotations of participants and stationary sound sources, other studies investigated dynamic effects of motion on speech intelligibility with moving sound sources around a listener (Davis et al., 2016; Pastore & Yost, 2017; Viveros Muñoz et al., 2019; Warnecke & Litovsky, 2021). It is important to consider such experiments in the context of speech intelligibility during self-rotation because of acoustical similarity with the self-rotation cases. Pastore and Yost (2017) investigated the effect of a moving target sound source, Viveros Muñoz et al. (2019) the effect of a moving interfering sound source, and Davis et al. (2016) considered both, a moving target and an interfering sound source. In the experiment of Davis et al. (2016), short target sentences were presented from either 0° or −60° such that either the target or distracter moved slowly towards the other but not both of them at the same time. The number of recollected key words of the target sentence improved in the moving conditions in comparison to the baseline stationary co-located conditions, indicating that the movement had a positive effect on the percent correct performance. However, the authors concluded that the improvement related to a change in spatial unmasking, which was shown by their Experiment 2. Pastore and Yost (2017) used single words in their speech-in-noise test. The distractor words were at ± 45°/ ± 90° symmetrically placed, and the target word was dynamically panned between −20° and 20°, panned at a single position of three possible in the given range, positioned at the center, or there was a co-located control condition. A small increase in intelligibility of the target word was found between dynamic and static panning, but it was attributed to a change of spatial unmasking in the dynamic condition. Viveros Muñoz et al. (2019) investigated the effect of a circularly moving interferer in a spatialized speech-in-noise task using German digit triplets as stimuli. They positioned the target in front of the listener either in an anechoic or a reverberant environment. The masker was either moving from the front 0° to 90° right, or it was positioned stationary at the midpoints of the moving masker stimulus as a means of controlling for the effects of dynamic changes of spatial unmasking during movement. In a group of older participants in the reverberant environment, the experimenters observed that the mean spatial release from masking decreased slightly, but significantly, in the moving condition, suggesting a negative effect of speech interferer movement on intelligibility. However, the decrease in the moving condition was not present in a group of younger participants. Warnecke and Litovsky (2021) investigated motion perception of chimera speech stimuli with varying intelligibility by manipulating the access to envelope cues. In the motion identification task, stimuli with more intelligible content and with speech-like envelopes were biased to be perceived more often as stationary, whereas sounds with noise-like envelopes and speech-like fine structure were more often perceived as moving. The study suggested that perception of auditory motion could be biased when signals involve cognitively salient content.

One possible mechanism for movement-induced effects on speech intelligibly is binaural sluggishness (Grantham & Wightman, 1978, 1979). The binaural system is known to limit access to binaural cues during quick binaural changes in the input signal. For instance, binaural unmasking effects for a tone in noise decrease when a relatively slow interaural temporal modulation (*f_m_* = 4 Hz) is imposed on the noise masker (Grantham & Wightman, 1979). This leads to assumptions of temporal integration of binaural cues on the order of hundreds of milliseconds, much larger than the typical high temporal resolution of the auditory system in other tasks, for instance, in silent gap detection it is only few milliseconds (Moore, 2012).

Although these previous studies considered moving target speech or moving interferers, the movement was restricted to relatively slow speeds. For instance, Viveros Muñoz et al. (2019) used speed of 32.73 °/s (circular moving masker), Pastore and Yost (2017) used 53 °/s. However, people naturally rotate at 150 °/s or even faster (Brimijoin et al., 2010). People move fast, especially when standing and are free to move their head and whole body to any orientation. Further, Hendrikse et al. (2018) investigated the effect of visual cues of target location on speech intelligibility, however, the targets were limited to the frontal field, which limited the extent of head movements. These methodological choices may explain why these studies did not observe effects of self-rotation on speech intelligibility, nor an effect of visual cues of location on speech intelligibility. Thus, the aim of the present study was (1) to investigate self-turning behavior of standing participants during listening to speech coming from an unexpected location; (2) to assess the effect of self-rotation on speech intelligibility in connection with changes in spatial unmasking due to self-rotation; (3) to assess the effect of presence of a visual cue indicating target location on self-rotating behavior and speech intelligibility. In order to study the dynamics of speech intelligibility, we used a sentence of five words as a target sound and measured intelligibility separately for each word. It was hypothesized (H1) that a visual indication of target location would help participants to rotate in an acoustically optimal way such that they obtain better speech intelligibility, (H2) that the changes in speech intelligibility due to the rotation would be closely related to changes in spatial unmasking due to the rotation, but that dynamic effects related to self-rotation would influence speech intelligibility in a negative way.

To address these hypotheses, a virtual acoustical representation of a reverberant room was simulated and auralized over loudspeakers in an anechoic chamber using the real-time Simulated Open Field Environment (Seeber & Wang, 2021). The free-field presentation was chosen to elicit realistic behavioral responses. We used target speech sources at azimuths that cover the whole circle around the participant and interfering sound in front of the participant to provide different degrees of dynamics in terms of the change of target-interferer configuration with different opportunities for motion-induced speech intelligibility benefit. During the experiment, the acoustic scene interactively adapted to the current horizontal angle of the participant, and therefore, the participants did not have to return to the initial position on every trial, which encouraged natural behavior. To make a more direct link to the spatial unmasking benefits during motion and speech intelligibility, a speech intelligibility model (Jelfs et al., 2011) and motion tracking were employed to predict intelligibility along the course of head orientation.

## Methods

### Participants

Young volunteers (*n* = 9, age: 26.6 ± 6 (median ± interquartile range), 1 female), native German speakers, took part in the study. Their hearing thresholds were checked with a calibrated audiometer (MADSEN Astera^2^, type 1066, Natus Medical Denmark Ap, Denmark). All pure-tone thresholds at standard audiological frequencies (250 Hz–8 kHz) were below or equal to 20 dB HL. One additional participant did not finish the study because of problems with hearing the target sounds in most conditions despite normal hearing thresholds and not reporting hearing problems. All participants provided written informed consent. Methodology and procedures were approved by the ethics committee of the Technical University of Munich (65/18S).

### Environment

The study was conducted in the Simulated Open Field Environment (SOFE v4) (Seeber et al., 2010; Seeber & Clapp, 2017). The SOFE v4 setup consisted of a high-fidelity sound reproduction system with a four-sided video projection inside an anechoic chamber (10 m × 6 m × 4 m: l × w × h). Thirty-six equally spaced active loudspeakers (Dynaudio BM6A mkII, Dynaudio, Skanderborg, Denmark) were positioned at 10° separation on a square-shaped construction (4.39 m × 4.39 m) at the height of 1.4 m and they pointed to the center of the square. The audio signals were played via a multi-channel sound card (RME HDSPe, Audio AG, Haimhausen, Germany) and digital-analog converters (RME 32DA, Audio AG, Haimhausen, Germany). The audio presentation system was calibrated, and loudspeakers equalized in frequency response, time of arrival and phase for frequencies between 100 Hz and 18 kHz to the array center point by a set of finite impulse response filters of 512 taps length at 44.1 kHz sampling frequency. The visual presentation system consisted of four high-resolution projectors (Barco F50 WQXGA, Barco, Kortrijk, Belgium) with low background noise (total of 32 dB(A) in the middle of the loudspeaker array) that project to four large acoustically transparent screens with projection area of 4.3 m × 2.7 m at distance of 2.15 m positioned right in front of the loudspeakers. The SOFE was further equipped with twelve high-speed optical motion-tracking cameras (OptiTrack Prime 17W, NaturalPoint Inc. Corvallis, Oregon, USA) which run in synchrony (eSync 2, NaturalPoint Inc. Corvallis, Oregon, USA) with the sound card via a word-clock signal. With the sound presented at 44.1 kHz, the motion tracking ran at 358.6 Hz such that each sample corresponded to 123 samples on the sound card. The experiment was controlled with three PCs using custom scripts written in MATLAB (v9.8.0 and v9.9.0, Mathworks, Natick, MA, USA) and Python (v3.6). The synchrony of the motion capture system and the sound presentation system was assessed by recording the in-ear signals of an artificial head (HMS II.3-33, Head Acoustics, Herzogenrath, Germany) which was rotated in the place of the participant. The motion trajectory was then used to re-create a ‘moving’ stimulus with a 0.5° resolution, which was recorded again by the static artificial head. We observed that interaural level differences between the two recordings were aligned.

The participants held a tablet touch-screen displaying all ten possibilities for each word of the OLSA (Wagener et al., 1999) sentences in matrix format, which could be tapped on. The GUI also displayed information on whether the participants could move or whether they should stand still. The GUI gave feedback on performance from the previous trial (i.e., the number of correct words out of five). Participants wore a motion-tracking crown, which was used to determine the position and rotation of the head. The position of the crown on the head was calibrated at the beginning of each experimental block (6 times during the experiment) to ensure precise measurement of self-rotations. The experimental program checked, at the beginning of each trial, if the participant was standing within 20 cm of the center of the loudspeaker array and in the Static condition it checked whether they were facing the frontal loudspeaker with a tolerance of 3°.

### Stimuli

The target sound stimuli consisted of twelve unique sentence lists from the OLSA matrix test (Wagener et al., 1999) presented at 60 dB SPL (at the participant's position) which were randomly assigned to each participant from a total set of 32 lists. Each list was fixed to one of four locations (0°, ± 90°, 180°) and one of three conditions (see below). OLSA lists consist of five-word sentences with fixed structure (e.g., ‘Britta gibt vier alte Bilder.’) such that each word was taken from a closed set of ten options. From each list, sentences 6–30 were used. The mean sentence duration was 2.19 s, the maximum sentence duration was 2.77 s, and the minimum sentence duration was 1.78 s.

The interferer sound was 4.5-s-long, and it always started one second before the target. It was stationary speech-shaped noise presented at 70 dB SPL with the same spectrum as the target sentence, which was computed for each sentence by taking the Fourier transform of the speech signal and randomizing the phase. Each token was ramped at the onset and the offset with a 50 ms Gaussian slope. The sound level of stimuli was defined as the level of the direct sound (the anechoic part without reflections) in the middle of the loudspeaker array at the listener position. The level was verified by a calibrated hand-held sound level meter (XL2, NTi Audio, Schaan, Liechtenstein) by measuring the level of speech-shaped noise played from one of the equalized loudspeakers.

**Figure 1.**
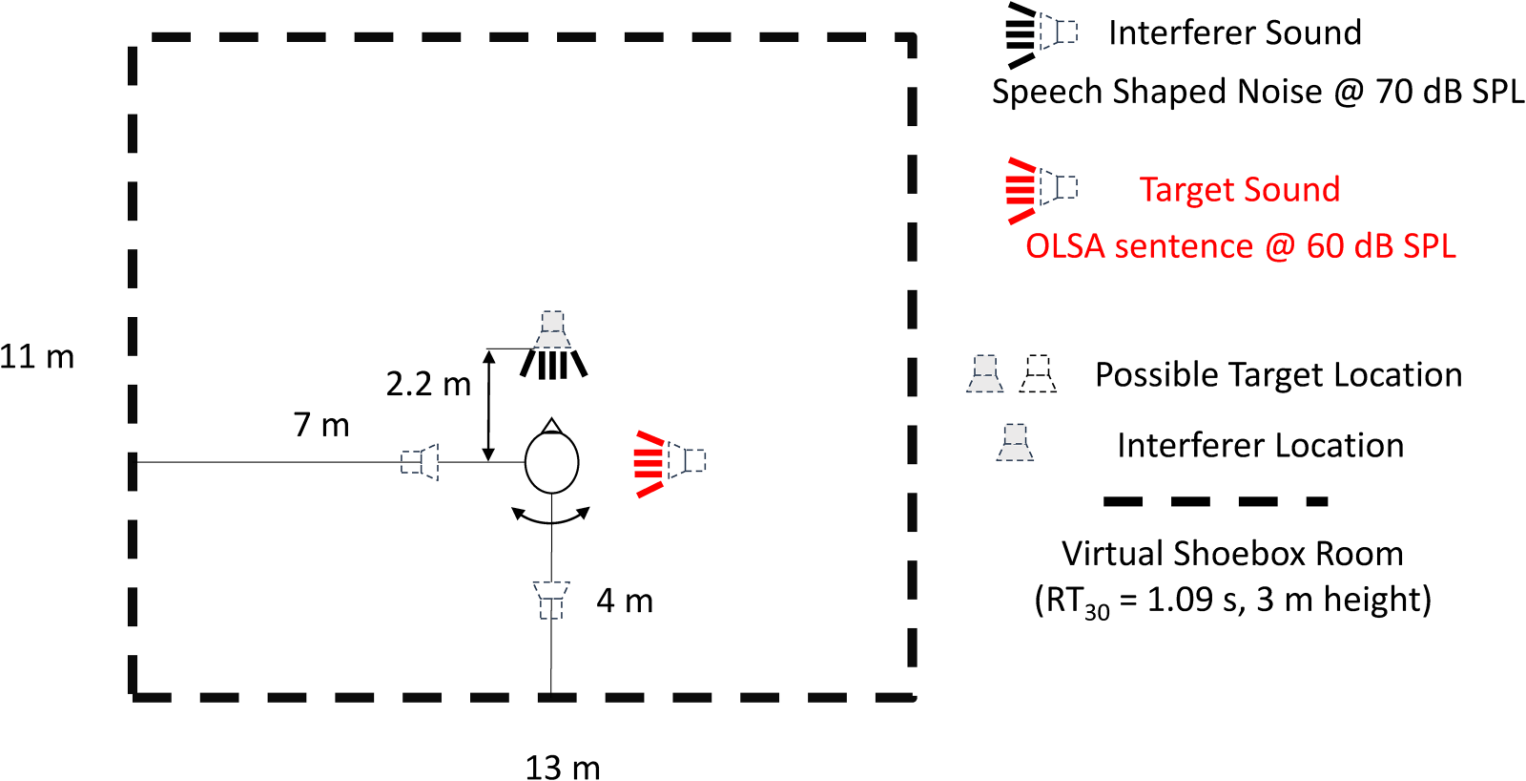
Position of the participant and of target and interferer sounds in the auralized virtual room.

All stimuli, targets and interferers, were spatialized in a virtual reverberant room over loudspeakers. Room acoustic simulation was used to create multichannel impulse responses for each SOFE-loudspeaker in the horizontal plane, which were convolved with the target sounds. This created reverberant conditions with sound sources distributed around the listener, which is shown in Figure 1. Dimensions of the virtual room were 11 m × 13 m × 3 m (l × w × h), and the virtual listener was placed off-center (4 m, 7 m, 1.8 m; l × w × h). The sound sources were positioned at 2.1 m distance from the listener. The acoustics of the virtual room was simulated using the image source method (Borish, 1984) up to the 100^th^ order with the real-time SOFE (Seeber et al., 2010; Seeber & Clapp, 2017), a freely available set of simulation and auralization tools (Seeber & Wang, 2021). Direct sound and individual reflections up to the fifth order were rendered to loudspeakers using the Ambisonics technique with max-rE weighting (Stitt et al., 2016). Higher-order reflections were mapped to the nearest loudspeakers. Reverberation time of the simulated impulse response (T_30_ = 1.16 s @ 250 Hz, 1.34 s @ 500 Hz, 1.15 s @ 1 kHz, 1.02 s @ 2 kHz, 0.85 s @ 4 kHz) was determined by ita_room_acoustics function of the ITA toolbox (Berzborn et al., 2017).

The visual stimuli consisted of a human-sized virtual character (*MakeHuman*, 201*9*) video-projected on the four screens surrounding the participant. The character appeared at the target azimuth synchronously with the onset of the target sentence and was visible until the start of the new sentence in the next trial. The synchrony of audio-visual experimental stimuli was assessed using a photosensitive LED and a pre-amplified measurement microphone connected to a storage oscilloscope (HMO724, Rhode & Schwarz). The analysis of 10 repetitions showed an offset of 80 ± 13 ms (mean ± std) between two stimuli which was accounted for in the experimental code.

### Conditions and Procedures

**Figure 2.**
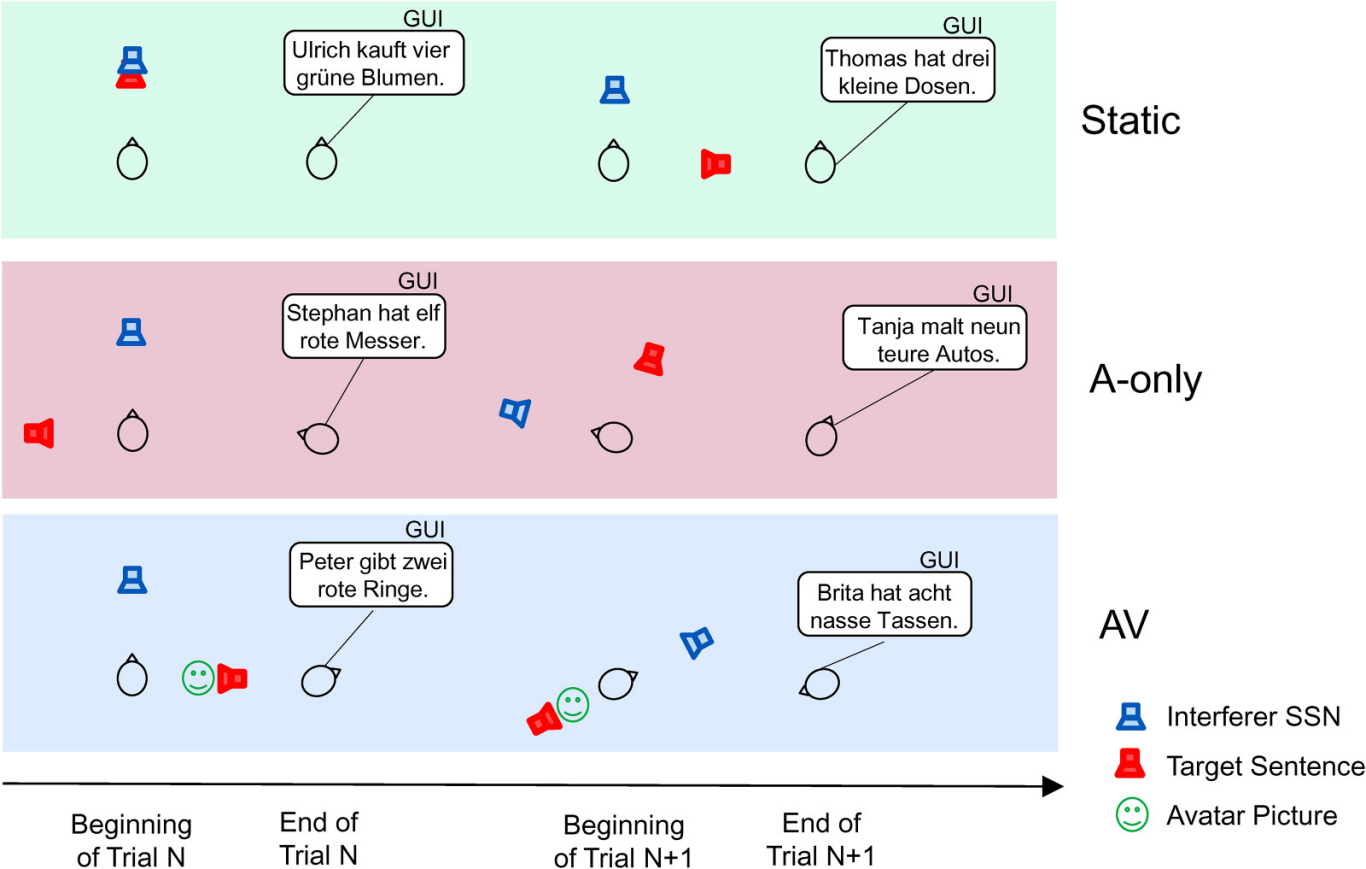
Experimental conditions. Each of the three conditions shows two example trials in a given block as a function of time. Presentation of the stimuli was followed by the behavioral response (usually self-rotation) and the response for the speech intelligibility task, the words understood were selected in a GUI. The interfering sound was always at the front of the person at the beginning of each trial, and the position of the target was chosen pseudo-randomly on each trial from one of four possibilities. In the AV condition, the target sound was accompanied by a picture of a virtual character.

The experiment involved three conditions: the Audio–Visual (AV), the Audio-only (A-only), and the static baseline (Static) (Figure 2). In all conditions, the target sentence (red loudspeaker in Figure 2) appeared pseudo-randomly on every trial at one of the four possible target locations (0°, ± 90°, 180° at 2.1 m from the listener), while the interferer was presented always from the frontal position (0° at 2.1 m from the listener). The acoustic simulation adjusted to the current participant's orientation at the beginning of each trial using the data from motion tracking of the participant. In the AV condition, the participants heard auditory stimuli and saw the virtual characters at the position of the target sound and they performed self-rotations. The A-only was identical to AV except the visual virtual character was not presented. The Static condition was identical to A-only except the participants were standing still and looking straight ahead in one predefined direction.

The participants were instructed to imagine a social situation in which somebody was talking to them. They were asked to listen to the target sentence, as if the approaching person was saying the sentence, and behave naturally. For instance, they could rotate toward the person, if it is something they usually do. After the sentence, the participants were asked to tap in the words of the presented sentence (each word had ten options) into the GUI on the handheld tablet and press the button ‘Weiter’. The new trial started shortly after the button press, and the program adapted to the current orientation of the person.

The GUI also displayed information about whether the participants could move or whether they should stand still. The GUI gave feedback on performance in the previous trials (i.e., the number of correct words out of five). When they did not understand a word, the participants were asked to choose an option randomly. The next trial could be initiated only if the participant chose five words, one from each category. Participants were told that in some blocks a virtual character would appear at the target direction while in other blocks there will be no virtual character, only sound. The participants were also told not to walk away from their position (participant could only rotate in place). In the Static condition, participants were instructed to stand still, listen to the target sentence, and provide a response to the GUI as in the other conditions.

The experiment was organized into six blocks with the condition fixed for each block. Each condition was presented in two blocks such that the first block involved the sentences from the first part of the sentence list, and the second block involved the sentences from the second part of the sentence list. The order of blocks was random and unique for each participant, with the limitation that the first three blocks involved all three conditions. Each block consisted of 48 trials; one trial consisted of one sentence presented together with interfering noise, followed by a response. Over the whole block, participants heard twelve sentences from each of the four possible positions.

Before the start of the main experiment, all participants underwent four training blocks. The training consisted of the blocks of the Static condition with the same procedures as described for the main experiment. However, only lists 33–40 from the OLSA CD were used during the training (these lists were not used in the main experiment), and the speech level was set to 64 dB SPL for the first two blocks, and 62 dB SPL for the second two blocks. For some participants, this was increased to 67 dB SPL and 63 dB SPL.

### Analysis

The behavior was analyzed in terms of horizontal self-rotations. The aim of the analysis was to determine whether the self-rotations were influenced by the presence of the visual cue of location (avatar picture) and whether participants rotate towards targets or towards acoustically optimal angles (see below). It was achieved by the quantitative and qualitative analysis of histograms of horizontal rotations during the final word of the target sentence and by the analysis of the predicted spatial unmasking using a speech intelligibility model (Jelfs et al., 2011) which defined the acoustically optimal angles as the angles that fell within 1 dB from the peak of the output of the model for all possible horizontal self-orientations. The data for the analysis were obtained from the motion tracking of the head of each participant. The raw data were rotated to align the motion tracking reference frame and the experimental reference frame. Then the data were transformed into Euler angles. The raw angular data of individual trials were referenced to the beginning of the trial, which corresponded to the interferer position, but due to the technical limitations (lag in the program), the interferer position was determined a few seconds earlier than the onset of the sound (see Discussion). The data were then unwrapped to avoid any discontinuities and smoothed with a 27 ms Kaiser window (twice, zero-phase, using MATLAB function filtfilt). In the next step, each individual trajectory was split into five sub-parts according to the duration of words in each sentence. The durations were determined by listening to all OLSA sentences (that were used in the experiment). The beginnings and the ends of all words in each sentence were manually labeled using a visual interface. In this way, precise temporal positions were obtained for each word of target sentence with respect to the rotation trajectory of each participant. From the pre-processed trajectories, the medians of the rotation angle for each individual word of the target sentence were computed. The data were analyzed using the toolbox for circular statistics (Berens, 2009).

Speech intelligibility was analyzed for each word of the target sentence. The analysis aimed to assess the difference in speech intelligibility due to the movement by comparing the A-only and the Static conditions, and whether speech intelligibility was influenced by the presence of the visual cue of the target location by comparing the AV and the A-only conditions. We used a balanced full-factorial design with factors of target location (four levels), condition (three levels), and word position (five levels) and the main outcome measure was the percentage of correctly answered words obtained from the pool of 24 answers. The percentage data were transformed using RAU (Studebaker, 1985) to obtain a more normally distributed pool of answers. The data were analyzed using the ANOVA model, implemented in CLEAVE (Herron, 2005). The ANOVA was run for the full model (three factors) and the planned contrast (two factors) (e.g., tests for the main effect of condition, word position, and their interaction separately for rear and lateral target directions). The reported p-values of the ANOVA were corrected using Geisser–Greenhouse Epsilons due to violations of the sphericity assumption in the within-subject designs. The effect sizes were reported in terms of the *η*^2^ (*r*^2^). The cumulative *η*^2^, the variance that can be explained by all source terms, is reported as *η_c_*^2^ only once per analysis.

To estimate the contribution of static spatial unmasking during motion (i.e., self-rotation influenced spatial unmasking), individual rotation trajectories were used in combination with the speech intelligibility model (Jelfs et al., 2011) implemented in the Auditory Modeling Toolbox (Søndergaard & Majdak, 2013) to predict intelligibility scores. The speech intelligibility model was designed to predict changes in spatial unmasking as shifts of speech reception thresholds in dB. However, in the current experiment the intelligibility was measured in % correct and the intelligibility scores covered almost the whole psychometric function, i.e., the intelligibility scores were measured above and the below threshold (50% intelligibility). Although the spatial unmasking could vary for different points of the psychometric function, we assume that such variation would not influence relative comparisons which are of interest in this study. Another speech intelligibility model (Beutelmann et al., 2010) was also tested for this purpose but since it provided almost identical results to the Jelfs et al. (2011) model, it was omitted from further analysis.

In order to apply the model to the current data, it was necessary to estimate the slope of the psychometric function and compute the speech intelligibility benefit due to head orientation (i.e., predict spatial unmasking) for each movement trajectory. The psychometric function was fit on the data from the Static condition using predictions of speech intelligibility in this condition (there were four target locations with different predictions by the model). We used the generalized linear random effect (GLRE) model implemented in MATLAB function fitglme to perform the fit. The head orientation benefit was computed by placing a virtual artificial head in the middle of the virtual loudspeaker array and virtually rotating the head according to the individual trajectory. This operation was performed using the multi-channel room impulse responses used in the experiment and the head-related transfer functions (HRTF) of the artificial head for all horizontal rotations and loudspeaker directions (measured in our anechoic chamber in 1° steps). Second, the median head orientation benefit in dB for each individual word was obtained. Third, the mean value across repetitions was computed and converted to a percent correct value using the fitted GLRE model (random intercept) and then transformed to RAU. The resulting average slope of the psychometric function was 0.08/dB.

The predictions were statistically compared to the intelligibility scores of participants using an ANOVA with pooled data of the AV and A-only conditions considering type of the data as a single factor.

## Results

### Self-Orienting Behavior During the Speech Intelligibility Task

**Figure 3.**
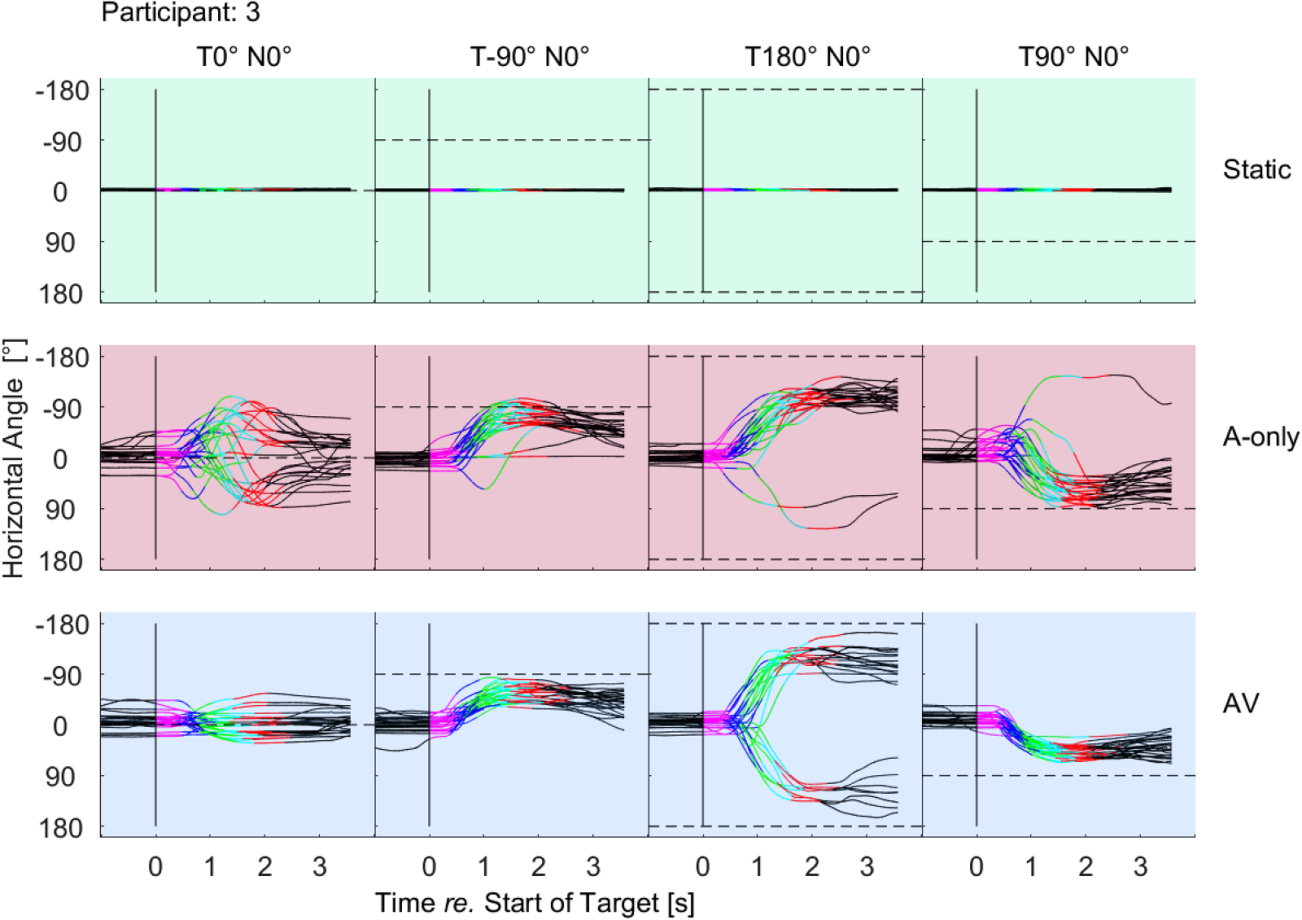
Self-rotation trajectories of one participant. The first row shows data for the Static, the second row for the A-only and the third row for the AV condition. The columns show data for different target locations, which are indicated by the horizontal dashed lines and the caption at the top that indicates the configuration of the target (T) and interfering noise (N). The colored parts of each line indicate the duration of each word of the OLSA sentence that corresponds to each trajectory. The black part represents the time when only the interfering noise was played. The beginning and the end of each trajectory correspond with the onset and the offset of the noise. The black vertical line at 0 s, indicates the onset of the speech signal.

Figure 3 shows the raw self-rotation trajectories of Participant 3. The participant was usually turning towards the target locations in the AV and A-only conditions and was standing still in the Static condition. The trajectories are characterized by an initial slow-movement phase (up to the target onset or shortly after the onset), an abrupt movement in the direction of the target, and the final corrective phase with a corrective rotation in the opposite direction of the initial rotation. Usually, this correction takes place towards the end of the target sentence (red part of the curves in Figure 3). While the earlier phases of the movement are determined by the target location and the onset of the target sentence, the corrective phase reflects the decision regarding the self-orientation for this particular situation. The end of the trajectory is usually offset from the target showing that the participant often undershot the target rather than pointed directly at the target.

**Figure 4.**
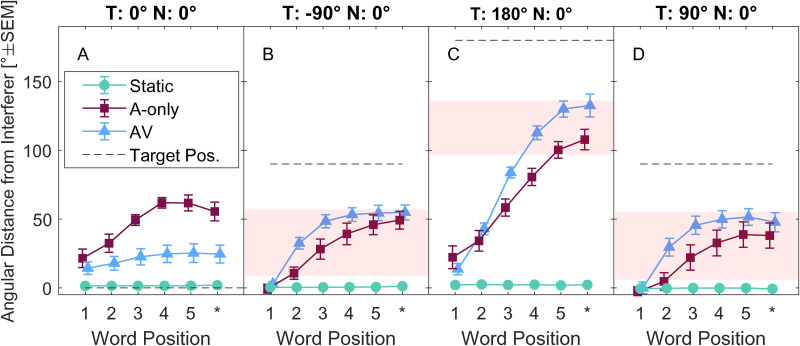
Across-subject averages of self-rotations unsigned angle differences relative to interferer position at 0° as a function of word position. For each participant, the data were computed as the across-trial mean of the unsigned azimuthal angular distance of the self-orientation for a given target word (five target words in each sentence plus a 500 ms period after the last word). The target location is depicted by a horizontal dashed line and changes in each of the panels A–D as indicated above. The red patches in panels C–D indicate the orientation range yielding the highest spatial unmasking, defined as within 1 dB from the maximum unmasking predicted by the speech intelligibility model (Jelfs et al., 2011), the acoustically optimal region. The star symbol ‘*’ indicates head angle position in the 0.5 s period after the end of the final word.

Undershooting is also visible in Figure 4, which shows across-subject means of self-orientation unsigned angles during individual words, thus depicting the evolution of self-rotation trajectories. The red-shaded area depicts predictions of acoustically optimal self-rotations, defined as within 1 dB from the maximum spatial unmasking according to the model. The symbols code the conditions: Static (•), A-only (▪), AV (▴); dashed horizontal lines show target locations. The acoustically optimal region for the frontal target is not considered because it was co-located with the noise source (negligible change of spatial unmasking).

There is a clear difference between AV and A-only (*F*(1,8) = 101.46, *p* < 0.001), indicating that the movements in AV were more pronounced towards the target locations. Further, self-orientations fell into the acoustically optimal region or close to it for most target words when the target was at the side (Figure 4B and D), and it fell into the optimal region only at the end of the target sentence for the rear target (Figure 4C).

**Figure 5.**
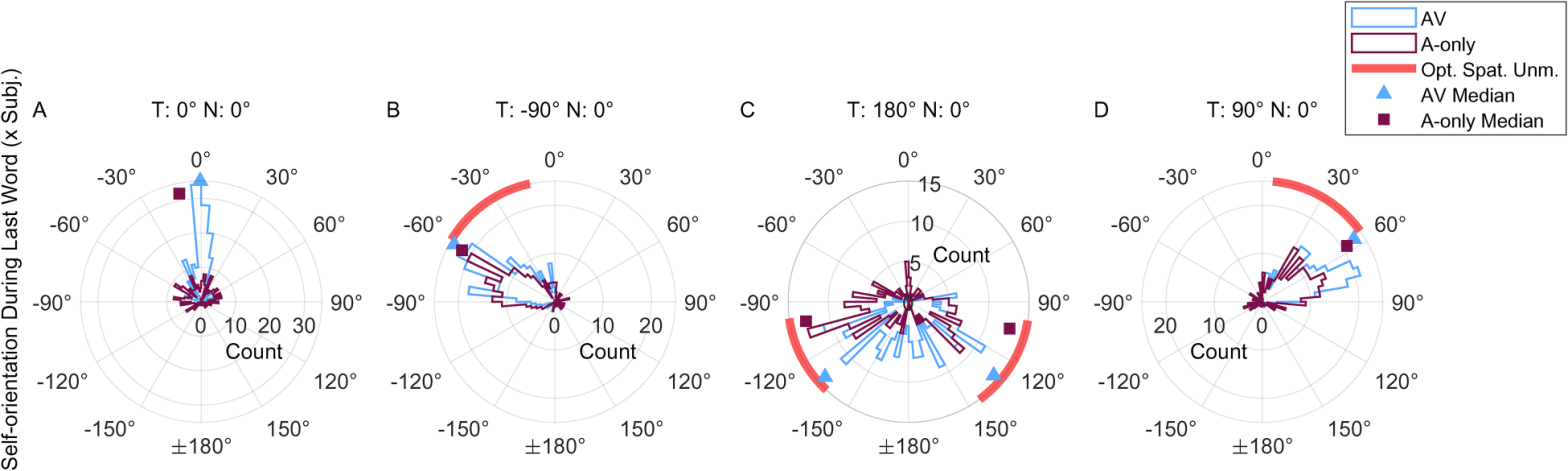
Rose histograms (5° bins) of self-orientation angles at the final word of the target sentence for the experimental conditions AV (blue) and A-only (burgundy). The histograms pool the raw data (median position during the final word) for each trial and participant. The data in panels A–D are organized according to the target location (written above each panel). The red stripes in panels C–D indicate the orientations with the highest spatial unmasking, defined as within 1 dB from the maximum unmasking predicted by the speech intelligibility model (Jelfs et al., 2011).

Figure 5 further investigates behavior on the last word of the sentence (Figure 4; Word Position 5). The data are shown in the form of across-subject distributions of head angles for the two conditions (AV- blue, A-only - burgundy) for each target location (panels A-D in Figure 5) and the data are statistically summarized in Table 1.

**Table 1.** Summary Across-Subject Statistics of Self-Orientation Angles During the Last Word of the Target Sentence, Rows Correspond to Different Target Locations (M – Data Mirrored).

		AV	A-only	AV	A-only	AV	A-only
	AV vs. A-only	Median re. Target (°)		Normalized Variance		Mean HOB (dB)	
T: 0° N: 0°	p: 0.001; k: 21600.0	−0.5	−11.5	0.180	0.592	N.A.	N.A.
T: −90° N: 0°	p: 0.010; k: 9072.0	27.4	29	0.142	0.306	4.07	3.20
T: 180° N: 0°	p: 0.001; k: 16200.0	−3.1	24.7	0.426	0.811	4.38	4.37
T: 180° N: 0° (M)	p: 0.001; k: 16200.0	49.4	74.1	0.133	0.213	N.A.	N.A.
T: 90° N: 0°	p: 0.005; k: 9720.0	−30.4	−33.4	0.134	0.365	3.71	3.04

The second column shows the p-value and the statistics of the two-Sample Kuiper test, which compares the two distributions from each panel in Figure 5. The third and the fourth column show the circular median in degrees for the two conditions. The fifth and the sixth columns show circular normalized variance (varies between 0 (tightly spread) and 1 (broadly spread)). The seventh and eighth column show predicted head orientation benefit (HOB) computed from the speech intelligibility model and the actual head orientation.

The distributions in Figure 5 substantially and significantly differ between AV and A-only (see Table 1). For the frontal angles in the AV condition (Figure 5A), the data are tightly grouped around the target location (at 0°), while they are spread in the A-only condition (see normalized variances in Table 1). For the lateral targets (Figure 5B and D), the data have similar overall patterns for the left-hand side target and, mirrored, for the right-hand side target. However, there is a higher spread of the orientation angles in the A-only condition than in the AV condition (compare A-only and AV normalized variances for the lateral targets in Table 1). Undershooting is similar for the lateral targets (approximately ± 30°, see Table 1). The data for the rear target are distributed in the rear part of the circle (Figure 5C); the A-only data follow a bimodal distribution which is symmetric around the (0°, 180°) axis, indicating that participants turned to the rear target leftwards and rightwards in a similar way. The bimodality is less clear for the AV condition where the data are more continuously spread. When the data are mirrored along the median plane, undershooting varies between ∼50° (AV) and ∼75° (A-only). The data still show a difference between AV and A-only in terms of normalized variance for the rear target. The mean head orientation benefit of speech intelligibility ranges from 3 dB (peak at 5.5 dB) for lateral targets to 4.4 dB (peak at 5.9 dB) for the rear targets (see Table 1). While these benefit values are slightly higher for the rear than for the lateral targets, and slightly higher in the AV condition than in the A-only condition, they are within 1.5 dB of the peak values.

### Speech Intelligibility During Self-Rotation

**Figure 6.**
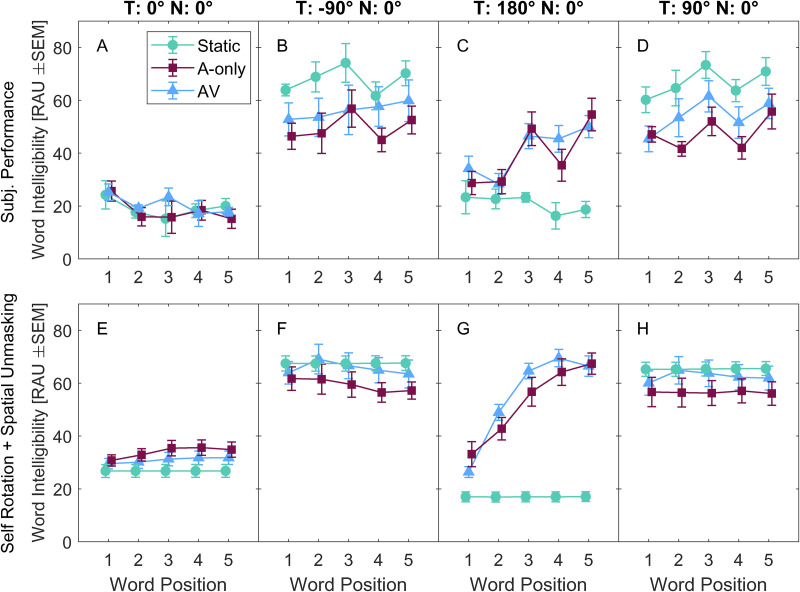
(A–D) Intelligibility of the individual words of the target sentence and (E–H) behavior-derived predictions using the speech intelligibility model (Jelfs et al., 2011) as a measure of contribution of spatial unmasking during the movement. The symbols determine the conditions with self-rotation (Blue triangles – AV, burgundy squares – A-only), the static baseline condition (green circles – Static). The abscissa shows the word position in the target sentence. Error bars indicate standard error of the means (SEM).

Figure 6A–D shows the across-subject mean speech intelligibility of individual words of the target sentence (x-axis). The bottom part of the figure (Figure 6E–H) shows predictions of speech intelligibility based on the motion trajectories, computed from the speech intelligibility model (Jelfs et al., 2011).

The variance in speech intelligibility (panels A–D) is driven mainly by the main effects of target location, condition, and word position and their interactions, and this is confirmed by a three-way within-subject ANOVA. Data of the left and right target locations are averaged into one level.

The ANOVA results are summarized in Table 2 and show that all main effects and interactions are significant. However, to further analyze the significant interactions, the dataset is split according to the target location and analyzed in two contrasts: Static vs. A-only to assess the effect of motion, AV vs. A-only to assess the effect of visual cues on speech intelligibility. The analysis focuses only on the lateral targets, and the rear target. This leads to 4 ANOVAs which are summarized in Table 3.

**Table 2.** Summary of Three-way ANOVA of RAU Transformed Speech Intelligibility Scores.

	F - statistics	p (corrected)	η^2^
COND	F(2,16) = 5.54	0.0240*	0.0071
WP	F(4,32) = 9.62	<0.0001***	0.0211
TARGET	F(2,16) = 121.48	<0.0001***	0.5049
TARGET*COND	F(4,32) = 43.35	<0.0001***	0.0903
TARGET*WP	F(12,96) = 5.86	<0.0001***	0.0257
WP*COND	F(8,64) = 2.83	0.0493*	0.0099
WP*COND*TARGET	F(16,128) = 2.75	0.0301*	0.0195

The included factors are condition (COND): A-only, AV, Static; word position (WP): 1–5; target position (TARGET): front, rear, left + right (averaged). The table further shows variance explained by the specific factors (η^2^) and variance explained by all terms (η_c_^2^).

Significance levels: * p < 0.05, *** p < 0.001 (η_c_^2^ = 0.6785)

**Table 3.** Summary of Four ANOVAs That Analyze RAU Transformed Speech Intelligibility Data for the Lateral and the Rear Targets Comparing Either Static vs. A-Only or A-Only vs. AV.

	F - statistics	p (corrected)	η^2^
Lateral target -Static vs. A-only (η_c_^2^ = 0.4226)			
COND	F(1,8) = 177.21	<0.00001***	0.3291
WP	F(4,32) = 7.42	0.0002***	0.0864
Rear target -Static vs. A-only (η_c_^2^ = 0.4858)			
COND	F(1,8) = 33.38	0.0004***	0.2643
WP	F(4,32) = 6.19	0.0051**	0.0864
COND * WP	F(4,32) = 6.26	0.0076**	0.1346
Lateral target - AV vs. A-only (η_c_^2^ = 0.1321)			
COND	F(1,8) = 10.25	0.0126*	0.05276
WP	F(4,32) = 5.12	0.0127*	0.0730
Rear target - AV vs. A-only (η_c_^2^ = 0.3829)			
WP	F(4,32) = 13.53	0.0010**	0.3594

The data from the left and right targets were averaged prior to this analysis. η_c_^2^ refers to variance explained by all terms of the ANOVA. Each repeated measures ANOVA was run with factors of condition (COND) and word position (WP).

Significance levels: *p < 0.05, **p < 0.01, ***p < 0.001

The results in Table 3 suggest that speech intelligibility is affected by movement and visual cues of location. The results show a significant difference between Static and A-only for the lateral and the rear targets. For the rear target, the difference between the conditions changes with word position. Further, the results show a significant difference between conditions AV and A-only for the lateral targets.

The modeled data from Figure 6A–D are analyzed to further assess the effect of visual cues of location on predicted speech intelligibility by comparing AV and A-only conditions for the lateral and rear targets using two ANOVAs. Next, the model predictions in the AV and A-only conditions are directly compared with speech intelligibility data in another ANOVA with one factor of data type (participant data, model data). The analysis is summarized in Table 4.

**Table 4.** Summary of Three two-way ANOVAs with Repeated Measures That Analyze RAU Transformed Model Predictions (see Text).

	F - statistics	p (corrected)	η^2^
Lateral target -AV vs. A-only (η_c_^2^ = 0.0776)			
COND	F(1,8) = 8.06	0.0218	0.0650
Rear target - AV vs. A-only (η_c_^2^ = 0.6828)			
WP	F(4,32) = 59.33	<0.00001***	0.6543
COND*WP	F(4,32) = 4.28	0.0330*	0.0216
Model vs. Participant Data (DATA TYPE) (η_c_^2^ = 0.3215)			
DATA TYPE	F(1,8) = 50.08	0.0001***	0.3215

The data from the left and right targets were averaged prior to this analysis. η_c_^2^ refers to variance explained by all terms of ANOVA.

Significance levels: *p < 0.05, **p < 0.01, ***p < 0.001

The analysis (Table 4) shows that the predicted speech intelligibility (based on self-motion) in the AV condition is statistically different from the A-only condition for both the rear and the lateral target. The effect size is, however, higher for the lateral targets than for the rear targets (compare η^2^ relative to η_c_^2^). The comparison of the predictions with participant data shows a statistical difference between these two datasets, the model overestimates participant data. The mean signed error is 11 RAU, which corresponds to approximately 1.4 dB according to the mean slope of the psychometric functions fitted from the Static data.

## Discussion

During the speech test, participants usually rotated themselves towards the target, away from the frontal interfering sound. The pattern of self-rotation varied for target positions and visual conditions. The self-rotations at the end of the test sentence were often close to what we defined as an optimal acoustic region (the region that falls within 1 dB from the maximum possible benefit). The mean head orientation benefits were in the interval between 3 dB (A-only) and 4.3 dB (AV), which is also very close to the maximum possible benefits of 5.5 dB and 5.9 dB, respectively (Table 1). Speech intelligibility in AV and A-only increased relative to Static for the rear target but not for the lateral or frontal target. Speech intelligibility was higher in AV than in A-only for the lateral target. Predicted speech intelligibility and participant data were closely related but the predictions overestimated participant data by approximately 1.4 dB.

### Self-Rotation Behavior

The observed self-rotations at the end of the target sentence systematically undershot the target and the undershooting increased with target laterality (29° for the lateral targets, 50°–75° for the rear target). Undershooting was not observed for the frontal target in the AV condition in terms of the median orientation (Table 1), yet the participants often maintained an off-target rotation (see raw data in Figures 3 and 4). Such undershooting is consistent with previous literature and it has been previously observed in various speech localization tasks (Brimijoin et al., 2010, 2012).

One possible explanation for the undershooting is that people employ a sound localization strategy. If people are unsure about the target location they scan the acoustic scene to find consistent sound localization cues of the target. Such scanning is evident in A-only for the frontal target (Figure 3) in which trajectories are more complex than trajectories in the AV condition and self-rotations at the end of the target sentence show higher normalized variance in A-only than AV, possibly because the target was more difficult to localize. Macaulay and Hartmann (2021) analyzed sound localization strategies of sitting people in a task with unrestricted head movements and showed that people integrate and weight localization cues to guide their behavior. In the present study, the task was to understand speech from various target locations and respond with self-rotation as if somebody was talking to the participant, which inherently includes both sound localization and speech perception. It is possible that people used a similar strategy for guiding behavior in the current experiment as in the experiments by Macaulay and Hartmann (2021). However, this search and localize behavior is likely to be dominant in the A-only condition, while the appearance of the visual cue might have triggered another strategy.

A possible strategy in the AV condition is a polite and effective communication strategy since people prefer to maintain an off-target self-orientation towards other people due to gaze aversion (Acarturk et al., 2021) while keeping access to visual cues for speech intelligibility. The undershooting is possibly beneficial acoustically because the ear points towards the target directly, a strategy that is robust in terms of spatial unmasking for different spatial distributions of noise sources (Grange & Culling, 2016). The polite strategy in the experiment is possibly related to spillover behavior (Galizzi & Whitmarsh, 2019), a learnt behavior that is beneficial in social context brought into the lab. Such behavior is shown even for the frontal target in the AV condition (Figure 3). Many trajectories show movement after the onset of the target which ended in slight undershooting.

Previous studies suggested that instructions were critical for behavioral patterns in this type of laboratory experiment (Grange et al., 2018). It was interesting to see that the behavior was more acoustically optimal under AV conditions than in the A-only conditions, and overall, not far from a possible optimum despite a lack of explicit instructions in the current experiment. Therefore, the information about the target location provided implicit cues for guiding behavior, which was reflected in less complex trajectories in AV than A-only (Figure 3) and lower normalized variance of head angles in AV than in A-only at the end of the target sentence (Table 1).

### Speech Intelligibility

#### The Effect of Motion

Speech intelligibility in the A-only condition decreased relative to Static for the lateral targets and increased for the rear target (Figure 6). The increase toward the rear can be accounted for by changes in spatial unmasking. The decrease cannot be easily explained, since model predictions based on self-motion did not predict the decrease for the lateral targets in A-only. Moreover, a constant offset of approximately 1.4 dB was observed between the modeled and the observed data in A-only and AV. A possible mechanism could be that the self-rotation limits the benefit of spatial unmasking due to binaural sluggishness (Grantham & Wightman, 1978, 1979), which can be modeled by applying an integration window after a fast cue extraction stage (Bischof et al., 2023). In this context, the speech model used in our study could be seen as a model without temporal limits, which extracts all cues optimally, a possible reason for it outperforming the participants. Another possible limitation of the employed speech intelligibility model is that the model was designed to predict spatial unmasking at the threshold (usually at 50% speech intelligibility), however, the model does not predict the slope of the psychometric function. Therefore, the unmasking predictions of the model at 20% or 80% of speech intelligibility might not be accurate.

Alternatively, attentional factors might be involved. For instance, recollection of what was being said could be limited due to the re-orientation in the scene and the realignment of the visual references. This possibility would be ruled out if the same experiment was repeated in virtual acoustic space without physical motion of the participants. Kondo et al. (2012) used such an approach in a streaming experiment. They showed that the self-rotation in an acoustic scene may lead to resetting of streaming. However, it was not the case when the self-rotation was performed in the virtual acoustic space without the physical rotation of the participant.

A small, possibly negligible, motion-related decrease in speech intelligibility was observed by Frissen et al. (2019) who studied the effect of speech-irrelevant head movements on speech intelligibility. The reason why they did not observe the negative effect, as in the present study, could be that they used multiple maskers in the acoustic scene whereas only one interfering noise source was present in the current study. This might have impacted the profile of spatial unmasking and the head orientation benefits. A negative effect of self-rotation on speech intelligibility was also not observed in Shen et al. (2017) who measured speech intelligibility during head rotation with seated participants. Although the data suggest a slight improvement in speech intelligibility for non-head turners relative to head turners, the difference was not significant. This could be related to a small spatial separation of the target and interferer sound, as well as to a small range of head movements in their experimental conditions.

#### Effect of Visual Cues of Target Location on Speech Intelligibility

The comparison of the AV and A-only conditions showed a change in speech intelligibility for the lateral target, but not for the rear target (Figure 6). This is likely attributable to the change of behavior between the conditions since the analysis of speech intelligibility predictions based on the rotation behavior showed similar trends, which suggests that the participants were likely to use the visual cue of location to alter rotation patterns for their benefit. The effect of visual cues was not observed for the rear target, this could be due to different behavioral patterns in lateral and rear conditions (the effect for the rear target was slightly smaller than for the lateral targets) or faster rotations in the rear target condition.

Hendrikse et al. (2018) observed that visual cues indicating the target location helped participants to identify the speech target out of multiple options, therefore they suggested prior knowledge of the target location could help participants orient in the scene. In our experiment, people were visually cued at the onset of the speech target while in Hendrikse et al. (2018) the target phrase started with a keyword and the target words were presented only two seconds later, which may have helped with attentional focus. In addition, they used multiple competing talkers as distractors, which might have required more attentional resources to focus on the target than the single continuous noise masker used in our study.

### Limitations of the Current Study

The speech intelligibility model employed in this study is a static model and is meant to provide a reference evaluation based on self-orientation as it does not consider dynamic effects. The model further assumes that the input speech signal is clean, without temporal fluctuations, and without reverberation. Reverberation in the target signal (used in the present study) reduces speech intelligibility, (Rennies et al., 2011). This may have affected the outcomes of the speech model; however, in our evaluations, we use relative comparisons, which would minimize this type of effect. Additionally, the model uses HRTFs recorded from an artificial head in situ in the experimental setup. Individualized HRTFs capture individual SNR differences, interaural cross-correlation differences and binaural cues with higher fidelity; however, the general trends should also be reproduced with non-individual HRTFs. In the analysis using HRTFs, we considered only horizontal rotations of the whole upper body (manikin), but shoulder reflections for different head orientations can alter interaural cues at higher frequencies substantially (Kolotzek, 2017). We do not assume that these acoustic effects would change the general outcomes of the study, but they could have contributed to the observed difference of 1.4 dB between psychoacoustically measured and predicted speech benefits.

In the experimental design, we aimed to align the orientation of the reference target location (0°) with respect to the listener always at the beginning of each trial. However, the participant's orientation was recorded about a second or two before the onset of the acoustic stimulus (due to a lag in the computer program), thus in some trials, people may have moved and were offset from 0° by the time of masker onset. The recorded angles were time aligned correctly in the analysis and in addition the behavioral data were re-analyzed without the three participants where this problem was the most prominent and this did not affect the results.

The acoustic stimuli involved sentence lists from a matrix test. Matrix tests use closed-set material, with rhythmic structure, lacking effects of context, prosody, or turn-taking cues; however, such material has been shown to generalize well to open-set material (Clopper et al., 2006), and it has been an established tool to study the effects of spatial unmasking, the focus of the present study.

The magnitude of spatial unmasking varied between target locations, however. While for the lateral and rear targets the mean predictions varied between 3 dB and 4.3 dB, for the frontal target the magnitude was negligible (but note a slight increase of AV and A-only relative to Static in Figure 6E). In acoustic environments with different profiles of reverberation and spatial configuration of the sounds, the magnitudes of spatial unmasking would be different which would have a direct impact on speech intelligibility and possibly on behavior, too. On the other hand, the current choice represents many common scenarios in mildly reverberant rooms.

## Conclusions

People self-rotated towards the speech target often in an acoustically optimal way defined as 1 dB from the maximum possible spatial unmasking. In the AV condition, participants rotated more often in the acoustically optimal way and the visual indication of target location helped them in terms of speech intelligibility in comparison to performance in the A-only condition for the lateral targets (H1). Speech intelligibility increased during self-rotations for the rear target but not for the lateral or frontal targets relative to a Static condition. Predicted speech intelligibility followed the trends but the ‘static’ model overestimated the participant data by approximately 1.4 dB, which suggests a negative effect of the dynamic motion on speech intelligibility, e.g., due to binaural sluggishness (H2).
